# Perinatal mental health around the world: priorities for research and service development in India

**DOI:** 10.1192/bji.2019.26

**Published:** 2020-02

**Authors:** Sundarnag Ganjekar, Anilkumar Viswananthan Thekkethayyil, Prabha S. Chandra

**Affiliations:** 1 Associate Professor, Department of Psychiatry, National Institute of Mental Health and Neurosciences, Bangalore, India; 2 Professor, Department of Psychiatry, Government Medical College, Thiruvananthapuram, India; 3 Professor, Department of Psychiatry, National Institute of Mental Health and Neurosciences, Bangalore, India. Email: chandra@nimhans.ac.in

**Keywords:** Perinatal psychiatry, India, LAMIC, postpartum depression, health systems

## Abstract

Maternal mental health disorders are a significant problem for mother–infant dyads in India, but have not received the attention that they should. However, recent major developments hold promise: the increase in coverage of the District Mental Health Programme; the growing emphasis in public health systems on newborn health; integration of maternal mental health into the Reproductive and Child Health Programme in the state of Kerala; and the Mental Health Care Act 2017, which mandates mother–infant joint care when a mother is admitted for mental illness, will lead to policy changes in services. Innovative implementation and translational research is needed to generate knowledge to strengthen maternal mental healthcare systems and improve maternal and child outcomes. Valuable ‘research rupees’ should be spent on ensuring equity of resources for physical and mental healthcare of mothers and providing optimal environments for every mother–infant dyad.

The priorities of India's Reproductive and Child Health (RCH) Programme have, thus far, been to reduce maternal and infant mortality and improve newborn health. A comprehensive public health programme for perinatal mental health is yet to evolve and there are very few dedicated perinatal psychiatry services. In recent years, several obstetricians, public health experts and psychiatrists have been advocating for prioritising perinatal mental health. The 2018 Biennial Scientific Meeting of the International Marcé Society in Bangalore was one such attempt, which helped put perinatal mental health in the spotlight.

Two major developments herald well for the future of perinatal mental health in India. First is the increasing coverage of the District Mental Health Programme, which will be able to provide an effective referral and training platform, and second, the growing emphasis in public health systems on newborn health. The government's National Health Mission has also started discussing ways of integrating perinatal mental health into existing mother and child programmes.

## What should our model for perinatal mental health services look like?

India is a large country, and, healthcare being under the control of individual states, there are wide variations in the provision of both mental healthcare and maternal healthcare. Therefore, it might be too ambitious to have a single plan that works countrywide. Bagadia & Chandra^1^ have proposed a stepped-care approach and the need to develop adequate care pathways. However, to do that, healthcare professionals first need to be sensitised to the importance and relevance of perinatal mental health. They should then be able to identify pregnant and postpartum women with mental health problems and also those who are at risk. The first step will therefore be to enhance the level of awareness of perinatal mental health among health professionals. Simultaneously, developing or adapting simple tools for detection will be a priority. Complex screening tools developed in the West often do not translate easily into Indian languages, have rating scales that are difficult for low-literacy women to complete and need to be validated for different groups.

Although screening and detection are important, they are useful only if identified mothers can be provided interventions. For this there is a strong need to train nurses, midwives and doctors in primary care, in both the public and private sectors, to provide suitable and evidence-based interventions and establish appropriate care pathways for more serious problems.

## *Amma Manasu* (Mother's mind) – the Kerala initiative

The state of Kerala has taken the lead in integrating mental health into routine antenatal and postnatal care and their model may give us some leads about country-wide implementation. A randomised controlled study (RCT) done in this state found a community-based depression intervention programme implemented in the public health setting to be effective in reducing severity of depression in women.^2^ Based on this experience, Kerala has now developed a state-wide maternal mental health programme, *Amma Manasu* (Mother's mind). Mothers will be assessed during their antenatal and postnatal visits by junior public health nurses, who will be trained to provide first-level interventions. Referral pathways will be established for stepped care and include doctors in primary care and the District Mental Health Programme. The Mother and Child Tracking System (MCTS) is an online data-entry platform used in India for all pregnant mothers and their babies. Couples are routinely sent information regarding nutrition, antenatal appointments and immunisation. It will be useful to use this technology both to identify mothers at high risk for mental health problems and to enable information-sharing between different stakeholders in the health system to provide seamless care for such mothers.

Based on the experience of the Kerala initiative, the National Health Mission is considering replicating the integrated maternal mental healthcare model into other states in India.

## Addressing other social determinants of poor maternal mental health

Two of the strongest predictors of maternal depression and anxiety are poverty and domestic violence. Programmes for maternal mental health will need to address these problems through involvement of policymakers, non-governmental organisations and effective use of laws.

## Need for mother and baby psychiatric units

For women with more severe mental illness, there is a strong need to develop mother and baby psychiatric units in district and teaching hospitals which are low resource and designed to meet cultural needs. The new Mental Health Care Act 2017^3^ mandates mother–infant joint care and the National Institute of Mental Health and Neurosciences (NIMHANS) mother and baby unit in Bangalore has shown that it is feasible to develop an Indian version of mother and baby units where families contribute to care-giving.^4^

## Research in perinatal mental health in India – the need for recognition, innovation and implementation

It is a matter of great concern that a recent expert consensus paper by the some of the largest research funding bodies in India highlighting the priorities for research in maternal, newborn and child health made no mention of mental health.^5^ This is despite the fact that the role of complex maternal conditions and fetal programming, as well as optimal environments needed for children's cognitive development, was emphasised. So, the first step will have to be to enhance research advocacy.

Indian research into perinatal mental health has focused so far on three areas: description, discovery and delivery.

### Descriptive studies

These have demonstrated the ubiquity of postpartum depression and anxiety and their relationship to social risk factors such as gender preference, social support, poverty and intimate partner violence as well as their impact on infant development. Clinical research has focused on psychopathology, suicidality, mother–infant bonding and patterns of help-seeking among mothers with more severe forms of mental illness.^6–8^

### Discovery studies

These have been few and have focused on the relationship between poor nutrition and maternal depression; there has also been limited translational research focusing on immune and genetic studies.^9,10^

### Delivery studies

There have been very few intervention studies. A low-intensity intervention for milder forms of depression, the Thinking Healthy Program Peer-delivered (THPP) trial in Goa, showed a moderate effect on remission from perinatal depression at 6 months postpartum and was found to be relatively cost-effective to deliver. Another study, conducted in rural North India, has shown that community mobilisation and participation may decrease rates of depression in addition to reducing maternal mortality.^11,12^

## How can we use our ‘research rupees’ more effectively?

**Fig. 1:**
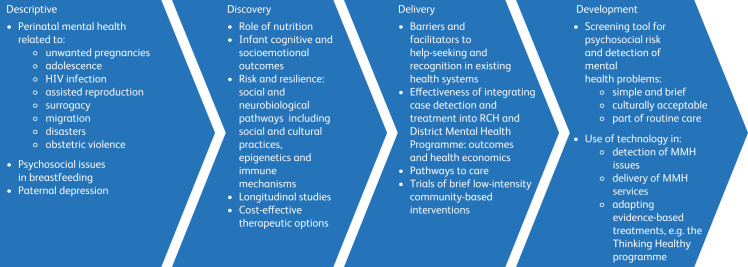
Research priorities for mother–infant mental health in India. RCH, Reproductive and Child Health Programme; MMH, maternal mental health.

We need to build on what is already done, think innovatively and focus both on implementation and translational research that will be worth the money and effort. The main goal of research should be to generate knowledge to strengthen maternal mental health systems and to improve maternal and child outcomes (Fig. 1). To this end we suggest the following top six strategies.

• Developing and validating simple screening methods that identify psychosocial risk factors and maternal distress.
• Understanding the magnitude and presentation of mental health problems in ‘high-risk’ groups, including adolescent mothers, mothers with poor perinatal outcomes, those with a history of mood disorders related or unrelated to previous childbirth, HIV infection, mothers facing childbirth trauma, including obstetric violence, mothers who have undergone assisted reproduction and migrant mothers, in order to develop targeted interventions.
• Identifying barriers and facilitators to accessing mental health services for maternal mental health problems, including explanatory models of perinatal mental illness among women and partners, the role of stigma and help-seeking patterns. We also need to understand what factors might enable women to access and accept mental health interventions.
• Understanding risk and resilience and the impact of mental illness on pregnancy and infant outcomes. There is limited research from India on the impact of psychosocial risk factors (such as depression, anxiety, stress and violence) in mothers on infant cognitive and socioemotional outcomes. In a social milieu such as India, where children often have alternative caregivers in addition to the mother, the role of protective factors in infant development is also not clear. Several cultural factors add to both risk and resilience. Risks include partner violence, patriarchy and gender preference, whereas protective cultural factors include rituals and infant-rearing practices. These may contribute to mother–infant interactions and maternal sensitivity. Fathers have been a neglected group and it is important to study men's role in the well-being of the mother–infant dyad. This will have direct relevance in identifying factors that contribute to risk and resilience and may inform interventions.
• Investing in translational research that may have a direct bearing on prevention and treatment of maternal mental health problems and their impact on the infant, including epigenetic studies, the role of nutritional supplements and immune studies. Establishing cohorts starting from pregnancy through infancy that combine psychosocial, developmental and biological data will be critical to our deeper understanding of the role of early adversities and protective factors.
• Conducting intervention and implementation research: we need to invest in testing brief and culturally suitable interventions for various aspects of mother–infant mental health. These may include screening and service-delivery models, parenting interventions, training and educational interventions and developing care pathways. When there are limited resources, outcomes need to be chosen carefully and should include acceptability, adoption, appropriateness, feasibility, cost, coverage and sustainability.

## Conclusions

Investing in perinatal mental health is important for preserving families and ensuring the physical and mental health of the coming generations. Policymakers and research funding bodies in India must ensure that there is equity of resources between the physical and mental health needs of mothers and provide an environment in which every mother–infant dyad has the best opportunity to thrive.
